# Maxadilan-simile expression in *Nyssomyia neivai*, a sandfly vector in an endemic region of Brazil, and its immunogenicity in patients with American tegumentary leishmaniasis

**DOI:** 10.1590/0074-02760160351

**Published:** 2017-02

**Authors:** Juliana Aires, Claudio Casanova, Sebastian Vernal, Margarida Nascimento, Sandra Rodrigues, Ethan A Lerner, Ana Maria Roselino

**Affiliations:** 1 Universidade de São Paulo, Faculdade de Medicina de Ribeirão Preto, Departamento de Clínica Médica, Divisão de Dermatologia, Ribeirão Preto, SP, Brasil; 2 Secretaria de Estado da Saúde, Superintendência de Controle de Endemias, Mogi Guaçu, SP, Brasil; 3 Universidade de São Paulo, Faculdade de Medicina de Ribeirão Preto, Laboratório de Sorologia, Hospital das Clínicas, Ribeirão Preto, SP, Brasil; 4 Universidade de São Paulo, Faculdade de Medicina de Ribeirão Preto, Departamento de Clínica Médica, Laboratório de Biologia Molecular, Ribeirão Preto, SP, Brasil; 5 Cutaneous Biology Research Center, Massachusetts General Hospital, Harvard Medical School, Boston, MA, USA

**Keywords:** Maxadilan protein, leishmaniasis, Psychodidae

## Abstract

**BACKGROUND:**

Maxadilan (Max) is a salivary component in the sandfly *Lutzomyia longipalpis* (Lutz & Neiva 1912), a vector of visceral leishmaniasis. Max has a powerful vasodilatory effect and is a candidate vaccine that has been tested in experimental leishmaniasis. *Nyssomyia neivai* (Pinto 1926) is a vector of the pathogen responsible for American tegumentary leishmaniasis (ATL) in Brazil.

**OBJECTIVE:**

We searched for Max expression in *Ny. neivai* and for antibodies against Max in ATL patients.

**METHODS:**

cDNA and protein were extracted from the cephalic segment, including salivary glands, of *Ny. neivai* and analysed by polymerase chain reaction, DNA sequencing, and blotting assays. The results were compared with data obtained from *Lu. longipalpis* samples. We quantified antibodies against Max in serum samples from 41 patients with ATL (31 and 10 with the cutaneous and mucocutaneous forms, respectively) and 63 controls from the endemic northeastern region of São Paulo state, using enzyme-linked immunosorbent assay.

**FINDINGS:**

Recognition of a Max-simile peptide by specific antibodies confirmed expression of a Max sequence in *Ny. neivai* (GenBank EF601123.1). Compared to controls, patients with ATL presented higher levels of antibodies against Max (p = 0.004); 24.4% of the patients with ATL and 3.2% of the controls presented anti-Max levels above the cutoff index (p = 0.014). The anti-Max levels were not associated with the specific clinical form of ATL, leishmanin skin test response, absence or presence of amastigotes in histopathologic exam, results of indirect immunofluorescence testing for leishmaniasis, or duration of cutaneous form disease.

**MAIN CONCLUSION:**

High serum anti-Max levels did not protect patients against ATL, but confirmed previous natural exposure to *Ny. neivai* bites in this ATL endemic region.

Leishmaniasis encompasses a spectrum of diseases caused by an obligate intramacrophage protozoan belonging to the genus *Leishmania* (Kinetoplastida: Trypanosomatidae). The parasite is transmitted by the bite of phlebotomine vectors (Diptera: Psychodidae: Phlebotominae) that inoculate promastigote forms of *Leishmania*, along with saliva, into the host’s skin while acquiring a blood meal (Roberts 2005). Pharmacological activities in the sandfly saliva promote blood feeding and cell functions, as well as modulate the host’s immune response (Champagne 1994).

Maxadilan (Max) is a 7-kDa peptide present in the salivary glands of the sandfly *Lutzomyia longipalpis* (Lutz & Neiva 1912). This species has been implicated in the transmission of *Leishmania* (*Leishmania*) *infantum chagasi* (Cunha & Chagas 1937), the main causative agent of visceral leishmaniasis (VL) in Brazil (Lainson & Rangel 2005). Max was the first molecule to be identified in sandfly saliva (Lerner et al. 1991), and it is a powerful vasodilator. Max inoculated into experimental animals exacerbates *Leishmania* infection, similar to inoculation of whole salivary glands (Morris et al. 2001). This peptide can redirect a Th1 response to Th2, up-regulating IL-10 and TGF-β production and suppressing IL-12p40, TNF-α, and NO production (Titus et al. 2006, Brodie et al. 2007).

The Max homologue in *Nyssomyia intermedia* (Lutz & Neiva 1912) (= *Lu. intermedia*), a vector of the pathogen responsible for American tegumentary leishmaniasis (ATL) in Brazil, is highly divergent - its shows only 34% sequence identity with Max from *Lu. longipalpis* and is less abundant (de Moura et al. 2013). The functional transcriptome of *Lu. ayacuchensis* (Cáceres & Galati 1988), a vector of cutaneous leishmaniasis (Kato et al. 2013), did not indicate the presence of Max in salivary glands. Hence, the amount of Max in the salivary gland of a vector may affect the outcome of *Leishmania* infection (Warburg et al. 1994).

Autochthonous cases of ATL have been reported in the northeastern region of the state of São Paulo. *Leishmania* (*Viannia*) *braziliensis* (Viana 1911) and *Leishmania* (*Leishmania*) *amazonensis* (Lainson & Shaw 1972) are the species associated with the cutaneous and mucocutaneous forms of ATL (Cruz et al. 2013, Neitzke-Abreu et al. 2014). However, to our knowledge, autochthonous cases of VL have not yet been reported in this region (Casanova et al. 2015).

The sandflies *Ny. intermedia* and *Ny. neivai* (Pinto 1926) (= syn. *Lu. intermedia*) are the main vectors of *Leishmania* (*V*.) *braziliensis* in the state of São Paulo. However, only *Ny. neivai* has been identified in systematic collections in the municipalities of that region (Andrade Filho et al. 2007). *Lu. longipalpis* is mainly found in the western region of the state of São Paulo, but it has not been associated with leishmaniasis in the northeastern region of this state (Casanova et al. 2015).

Our objective was to verify the presence of antibodies against Max in patients with ATL, because it has been studied mainly in animal models and only rarely in humans. First, in this study we demonstrated Max-simile gene and protein expression in *Ny. neivai*. Then, we compared anti-Max IgG antibody levels in autochthonous cases of ATL to those in healthy controls, which confirmed exposure to bites of the *Ny. neivai* vector in the northeastern region of São Paulo state, Brazil.

## MATERIALS AND METHODS

*Maxadilan* - The recombinant peptide was produced in *Escherichia coli* as described previously (Lerner & Shoemaker 1992).

*Rabbit immunisation against Max peptide* - Briefly, 100 mL of Max (150 mg/mL) was diluted in phosphate buffered saline (PBS) to a final volume of 2,000 mL and distributed into 500-mL aliquots. On the first day, 500 mL of complete Freund’s adjuvant (Sigma-Aldrich, Saint Louis, MO, USA) was mixed with an aliquot of Max, and a rabbit was injected with the mixture in each hip. Nine days later, another dose was injected in the foreleg and shoulder of the rabbit. On day 20, a third dose was injected at four points subcutaneously. Rabbit serum was tested by counterimmunoelectrophoresis on a 1% agarose gel before and after immunisation (positive pole) against Max (negative pole). Runs were performed using sodium barbital buffer in an electric current of 30 mA for 60 min, and then agarose gel was stained with Coomassie Blue (Sigma-Aldrich, Saint Louis, MO, USA).

*Sandflies and RNA-DNA-protein extraction* - Laboratory-colonised *Lu. longipalpis* were provided by Alda Hawk, PhD, Fiocruz-Belo Horizonte, state of Minas Gerais, Brazil; *Ny. neivai* specimens were wild caught and identified by Claudio Casanova, SUCEN-Mogi Guaçu, state of São Paulo, Brazil. The cephalic segments, including the salivary glands, of *Lu. longipalpis* and *Ny. neivai* specimens were used for extraction by the Trizol method. In this report, the sandfly species names are presented in accordance with Galati’s classification system (Galati 2003) and, when cited for the first time, are followed by the corresponding nomenclature of Young and Duncan in brackets (Young & Duncan 1994). Abbreviations of generic names follow the proposal of Marcondes (2007).

*Protein electrophoresis* - Max (0.33 mg/mL) and proteins extracted from *Lu. longipalpis* (0.02 mg/uL) and from *Ny. neivai* (0.01 mg/mL) were run in a 12.5% acrylamide gel using a Mini Protean II (Bio-Rad, Hercules, CA, USA*)*; the acrylamide gel was stained by the silver method.

*Immunoblotting* - Max and protein extracts submitted to electrophoresis were transferred to a 0.2-mm nitrocellulose membrane (Bio-Rad, Hercules, CA, USA) (Mini Trans-Blot Cell, Bio-Rad, Hercules, CA, USA). Then, each strip was incubated with a serum sample (1:20) for 2 h. After washing with thiosulphate-citrate-bile salts-sucrose (TCBS), a protein G-horseradish peroxidase (HRP) conjugate was incubated with the strips for 2 h, which was followed by colour revelation with an HRP-conjugated substrate (Bio-Rad, Hercules, CA, USA).

*Comparison of the Ny. neivai* - Max DNA sequence with that of *Lu. longipalpis* using polymerase chain reaction-restriction fragment length polymorphism (PCR-RFLP). The Max DNA fragment was amplified with specific primers (Invitrogen, Carlsbad, CA, USA): sense-GCCATAGATGACTGCCAGAAGC and antisense-TTCCAGGTAGTTGGGAGGTATCC. PCR was performed in a thermocycler (Thermo Fisher, Waltham, MA, USA) with a final volume of 25 mL: 2.5 mL of 10 × buffer, 5 mL of 2 mM dNTP, 10.3 mL of distilled water, 1 mL 100 pmol of each primer, and 20 mL of Taq polymerase. A total of 35 cycles were run as follows: one cycle at 93ºC for 30 sec, one cycle at 94ºC for 3 min and 30 sec, one cycle at 52ºC for 1 min, one cycle at 72ºC for 1 min, and a final cycle at 72ºC for 10 min. Amplification of a 106-bp product was confirmed in a 10% acrylamide gel. *Hha*I and *Rsa*I enzyme restriction was performed. *Hha*I acts at the GCG^C restriction site, generating 30-bp and 80-bp sequences. *Rsa*I acts in GT^AC, resulting in two 50-bp sequences.

*Reverse transcription-PCR (RT-PCR)* - RNA (12 µL) extracted from both sandflies species was added to 0.7 mL of RNase inhibitor, 4 mL of 5× RT buffer, 1 mL of 10 mM dNTP, 0.6 mL of RT (Invitrogen, Carlsbad, CA, USA), and 1.7 mL of distilled water at 42ºC for 90 min, followed by storage at -20ºC. cDNA was used in the PCR assay described above.

*Sequencing of PCR products* - PCR was performed with the sense primer-CGTGTTTGCCTTCAGTAAGTTCT to amplify a long fragment and for sequencing. A 246-bp fragment was sequenced following the manufacturer’s recommendations (DNA Sequencing Kit Big Dye Terminator, Applied Biosystem, Foster City, CA, USA; and ABI Prism 310 Genetic Analyzer, Applied Biosystem, Foster City, CA, USA).

*Study population* - Patients were identified at the University Hospital, Ribeirão Preto Medical School, University of São Paulo, Brazil, which is the main reference institution for diagnosis and treatment of ATL in the northeastern region of the state of São Paulo. A diagnosis of ATL was confirmed with at least two of the following criteria: clinical-epidemiological diagnosis compatible with ATL; positive leishmanin skin test (LST; Adolfo Lutz, Brazil) defined as induration ≥ 5 mm 48-72 h after injection; histopathology of a skin or mucosa sample indicative of ATL, with or without visualisation of the amastigote form; indirect immunofluorescence (IIF) test (Fiocruz, Brazil); and PCR result positive for the *L. Viannia* subgenus (Vernal et al. 2016). All patients were negative for anti-HIV antibodies.

Patients’ serum samples were selected randomly from a serum bank at the Laboratory of Dermatology, University Hospital. The control group consisted of 63 healthy donors selected randomly at the Ribeirão Preto Blood Center.

*In-house enzyme-linked immunosorbent assay (ELISA) standardised with Max peptide* - Briefly, Max diluted in carbonate-bicarbonate buffer (125 mg/mL) was used to coat a microplate (Immulon, Thermofisher, Waltham, MA, USA) at 4ºC for 48 h. After washing with PBS-Tween 20 (T20), 50 mL of each serum sample diluted in PBS-TM is Phosphate-buffered saline (PBS) plus Tween 20 plus Milk, plus 5% fat free milk (1:50) was incubated at 37ºC for 2 h. After washing, peroxidase-conjugated anti-human IgG (Sigma-Aldrich, Saint Louis, MO, USA) was incubated with plates at 37ºC for 2 h, and 100 µL of chromogenic substrate (*o*-phenylenediamine dihydrochloride, Sigma-Aldrich, Saint Louis, MO, USA) was incubated with plates at room temperature in the dark for 15 min, followed by the addition of 50 mL of stop solution (1N H_2_SO_4_). Colour development was observed in an ELISA reader (Labsystem Multiskan MS, Artisan, Champaign, IL, USA) at 492 nm. Anti-Max values were expressed as index values: the optical density (OD) of each sample was divided by the cutoff (the mean anti-Max OD values in the control group plus two standard deviations).

*Statistical analysis* - Patients’ data are summarised the table. The Student’s *t-*test was used to compare the results of two groups. Categorical variables were compared by Chi-square (χ^2^) test. Fisher’s exact test was used when data were sparse. Significance was set at p < 0.05; a two-tailed comparison was employed. The statistical analyses were performed using GraphPad Prism 6 software.

**TABLE:** Demographic and laboratory data from patients with cutaneous and mucocutaneous clinical forms of American tegumentary leishmaniasis

	Cutaneous form	Mucocutaneous form	p*-value*
	
	Median (min/max)	Median (min/max)	
	
Age (years)	44.0 (3.0/64.0)	51.5 (22.0/65.0)	0.560

Duration of disease (months)	4.0 (1.0/31.0)	66.0 (4.0/300.0)	< 0.001

	N (%)	N (%)	
Gender			
Male	23 (74.2)	10 (100.0)	0.083
Female	8 (25.8)	0 (0)	
Leishmanin skin test			
Positive	18 (64.3)	7 (77.8)	0.376
Negative	10 (35.7)	2 (22.2)	
Presence of amastigotes^a^			
Present	9 (29.0)	4 (40.0)	0.390
Absent	22 (71.0)	6 (60.0)	
IIF			
Reactive	18 (58.1)	6 (60.0)	0.606
Non-reactive	13 (41.9)	4 (40.0)	
PCR			
Positive	18 (58.0)	4 (40.0)	1.00
Negative	10 (32.3)	3 (30.0)	
Not done	3 (9.7)	3 (30.0)	

*a*: on histopathological biopsy sample; IIF: indirect immunofluorescence; min: minimum; max: maximum; N: number of patients.

*Ethics* - This study was approved by the Human Ethics Committee (#5.886/2002) of the University Hospital of the Ribeirão Preto Medical School, University of São Paulo, Brazil, in accordance with the ethical standards of the Helsinki Declaration (1964, amended most recently in 2008) of the World Medical Association. All patients included in the study signed to indicate their informed consent prior to participation.

## RESULTS

**Fig. 1:**
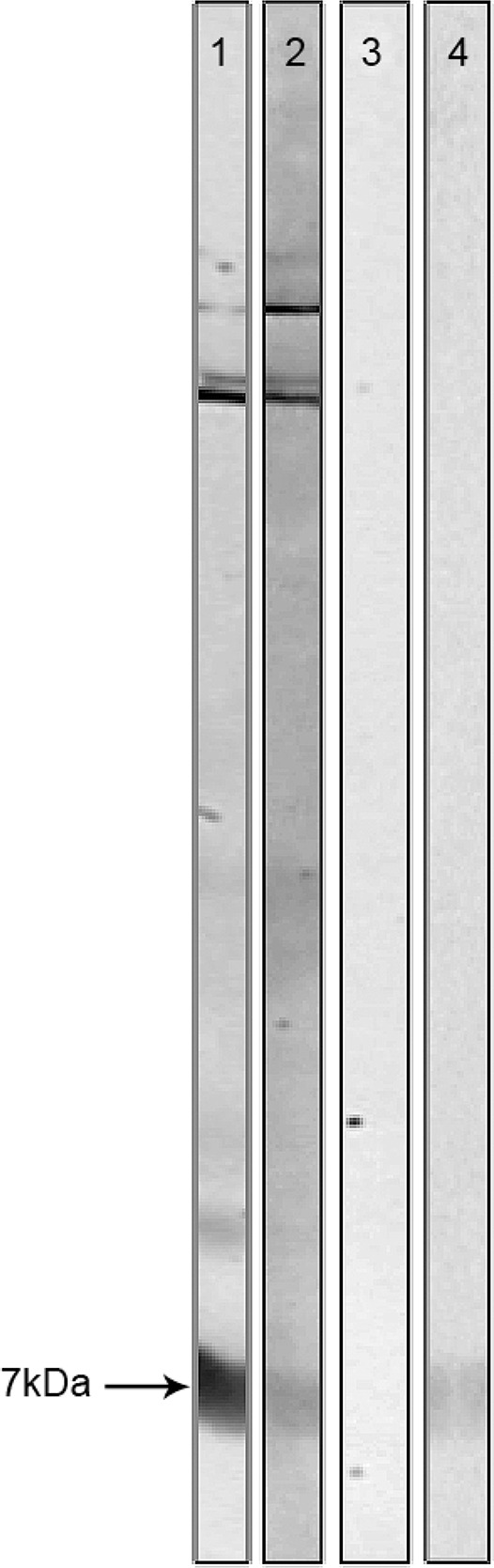
: immunoblotting with the protein extracts from *Lutzomyia longipalpis* (strip 1 and 3) and *Nyssomyia neivai* (strip 2) and from Maxadilan (Max) recombinant protein (strip 4). Strips 1 and 2 were incubated with anti-Max rabbit serum diluted 1:20; strip 3 was incubated with rabbit serum before immunisation diluted 1:20; strip 4 was incubated with anti-Max rabbit serum diluted 1:20. Anti-Max serum recognised a 7-kDa peptide (arrow).

*Immunoblotting indicated Max-simile peptide in the Ny. neivai protein extract* - Similar molecular weight fractions, including a 7-kDa peptide, were present in both the *Ny. neivai* and *Lu. longipalpis* protein extracts (data not shown). Immunoblotting with rabbit serum following immunisation against Max and Max peptide confirmed the immunisation specificity observed in the counterimmunoelectrophoresis. Subsequently, Max-immunised rabbit serum incubated with *Ny. neivai* protein extract confirmed a protein fraction with a molecular weight similar to that of Max (Fig. 1).

**Fig. 2:**
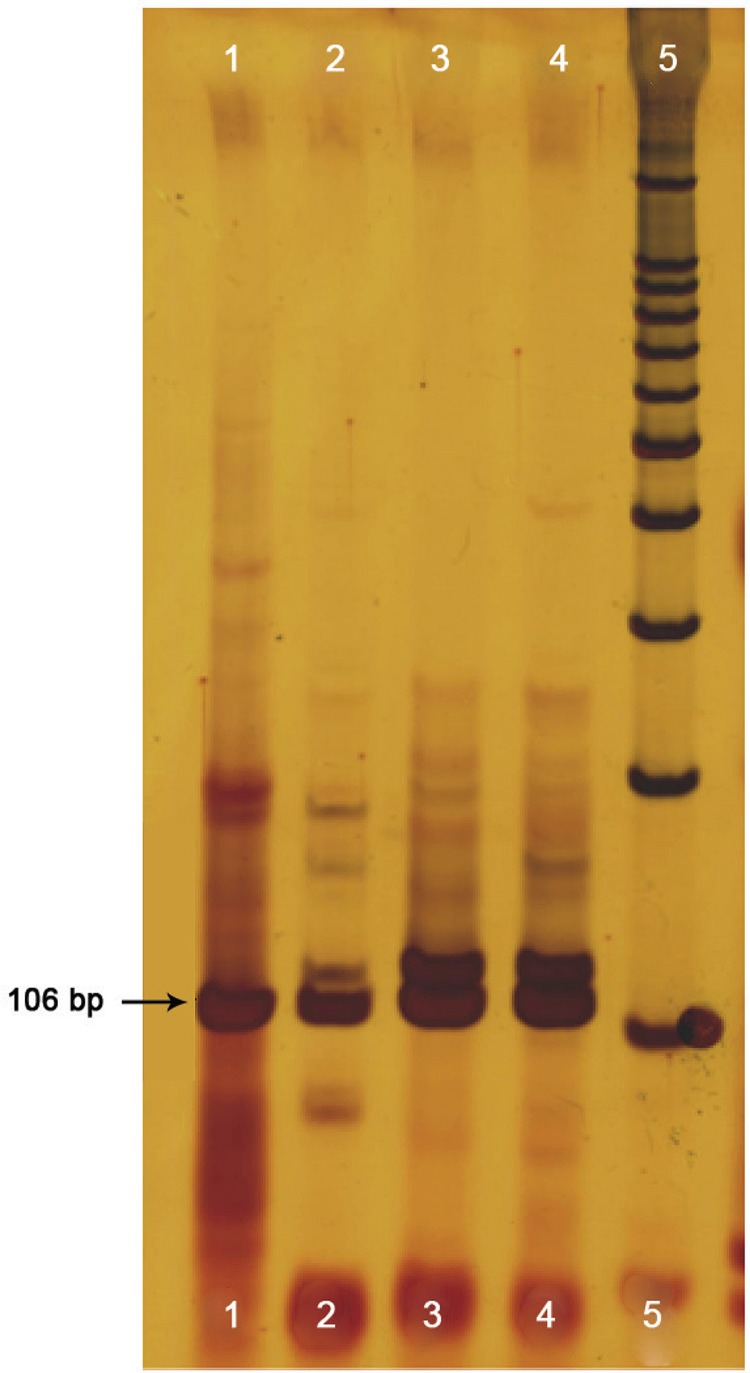
: a 10% acrylamide gel showing 106-bp amplicons (arrow) using specific primers for Max. (1) DNA from *Nyssomyia neivai*; (2) cDNA from *Ny. neivai*; (3) DNA from *Lutzomyia longipalpis*; (4) cDNA from *Lu. longipalpis*; (5) molecular weight marker = 100 bp.

**Fig. 3:**
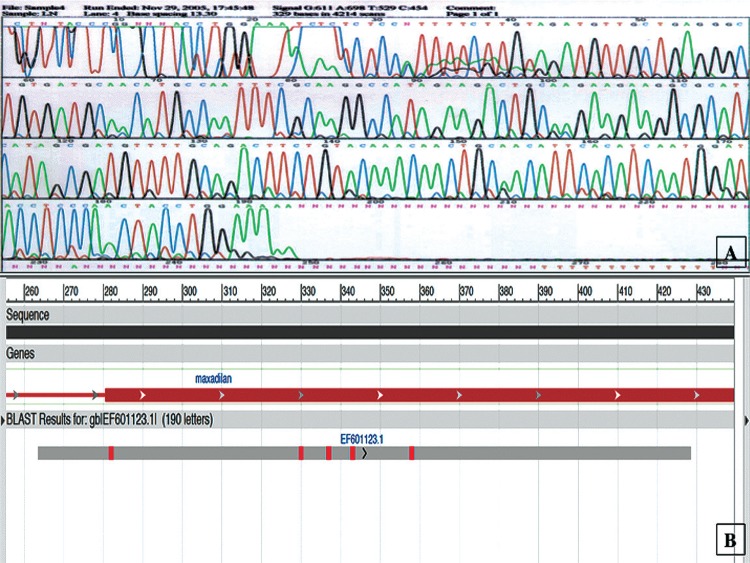
: the cDNA Maxadilan sequence is expressed in *Nyssomyia neivai* and was aligned with Maxadilan of *Lutzomyia longipalpis*. (A) cDNA sequencing confirmed a Maxadilan-simile fragment in *Ny. neivai* (gi|148565454|gb|EF601123); (B) alignment of a Maxadilan sequence from *Lu. longipalpis* (gi|159451|gb|M7790.1|LUTMAX) and other *Lu. longipalpis*. Available from: http://www.ncbi.nlm.nih.gov/, accessed June 30, 2016.

*PCR-RFLP and DNA sequencing indicated Max expression in Ny. neivai* - PCR-RFLP and DNA sequencing revealed a 106-bp amplicon from *Ny. neivai* extracts resembling the amplicon observed from *Lu. longipalpis* extracts. Enzymatic restriction with *Hha*I and *Rsa*I showed a similar restriction pattern in both samples (Fig. 2). DNA sequencing of a 248 bp-amplified fragment showed 66.5% Max gene homology (92% identity and 100% similarity with the Max gene). This DNA sequence was deposited in GenBank (accession no. gi|148565454|gb|EF601123) (Fig. 3).

*Anti-Max values and ATL disease* - Forty-one patients with ATL participated in the study (31 and 10 patients with the cutaneous and mucocutaneous clinical forms, respectively). The patients’ median age was 45 years (minimum of three-years-old and maximum of 65-years-old); 80.5% of the patients were male. The median duration of the disease in the patients with ATL was six months (minimum of one month and maximum of 300 months). The time for disease evolution was greater in patients with the mucocutaneous form than in patients with the cutaneous form of ATL (median of 66.0 months and 4.0 months, respectively; p < 0.001) (Table).

**Fig. 4:**
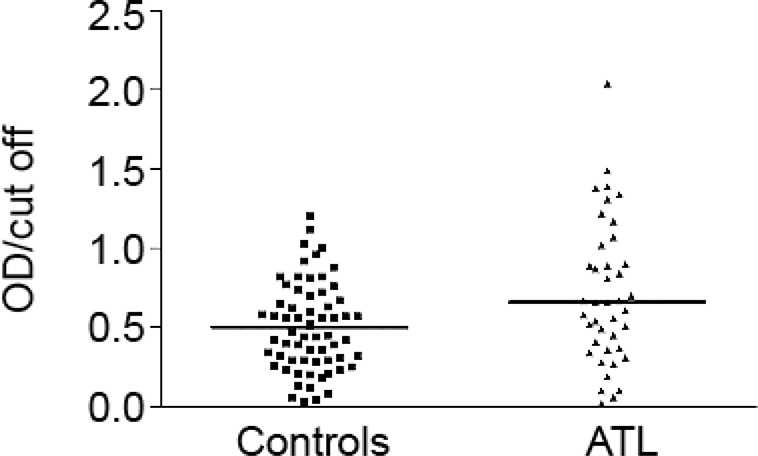
: antibodies against Maxadilan (presented as index values) identified by enzyme-linked immunosorbent assay in American tegumentary leishmaniasis (ATL) cases and the control group. Control group versus patient with ATL (p = 0.0043). Index values were calculated from the OD (optical density, 492 nm) value of each sample divided by the cutoff OD value. Cutoff: median OD values in the control group plus two standard deviations.

Anti-Max antibodies levels (presented as index values) were higher in the ATL patients than in the controls (p = 0.0043) (Fig. 4). Based on a cutoff value of 1.0606, 10/41 (24.4%) of the patients with ATL and 2/63 (3.2%) of the controls presented anti-Max levels above the cutoff (p = 0.0014). Anti-Max levels were not associated with the clinical form of ATL (p = 0.7044), LST response (p = 0.8440), absence or presence of amastigotes in the histopathologic exam (p = 0.4697), IIF levels (p = 0.5893), or duration of the disease (0-4, 4-8, > 8 months) in the cutaneous form (p = 0.2969).

## DISCUSSION

Over the last few decades, the number of ATL cases has been increasing worldwide. In the Americas, the number of ATL cases has grown markedly, and Brazil has contributed the majority of the cases (Alvar et al. 2012). The state of São Paulo is not an exception: the number of autochthonous ATL cases has increased, and the areas where cases are reported have expanded (SES-SP 2008). Sandflies that were formerly restricted to rural areas are now widely distributed across this state (Andrade Filho et al. 2007, Casanova et al. 2015).

*Lu. longipalpis* has not been identified in the northeastern region of the state of São Paulo, and to our knowledge, autochthonous cases of VL have not been reported in this region (Casanova et al. 2015). In the same way, *Ny. intermedia*, which is a vector of ATL in the state of São Paulo (Andrade Filho et al. 2007) with a Max homologue detected in its salivary gland transcriptome (de Moura et al. 2013), has not been identified in this region (Andrade Filho et al. 2007). Therefore, we decided to investigate whether *Ny. neivai*, a sandfly vector of the pathogen responsible for ATL in the region, also harboured Max. Our results have shown that the Max gene is expressed in *Ny. neivai* (GenBank EF601123.1) and that this fragment shares 66.5% identity with Max from *Lu. longipalpis*.

Regarding the *Ny. intermedia* transcriptome, its Max homologue is very divergent (34% identity) and the protein is present in low amounts compared to Max in *Lu. longipalpis*. As for *Lu. ayacuchensis*, another vector of cutaneous leishmaniasis in the New World, its transcriptome does not show Max gene expression, but large amounts of other vasodilator molecule genes such as adenosine and AMP are expressed (Kato et al. 2013).

Although blotting confirmed Max-simile peptide expression in the protein extracts of *Ny. neivai*, the amount of this salivary protein deserves further investigation. Even though the cephalic segment, including the salivary glands, extracted from sandflies may have contained several contaminants for the blotting reaction, specific rabbit antibodies against Max recognised a 7-kDa peptide.

Mechanisms that enhance *Leishmania* infection in sandfly saliva are associated with its immunomodulatory properties (Titus & Ribeiro 1988, Kamhawi 2000, Andrade et al. 2007). In experimental models, saliva from *Lu. longipalpis* exacerbates infection caused by *Leishmania* (*V.*) *braziliensis* and *Leishmania* (*L.*) *amazonensis* when the saliva is inoculated together with the parasite (Samuelson et al. 1991, Laurenti et al. 2009). In contrast, saliva of wild-caught *Bichromomyia flaviscutellata* (Mangabeira, 1942) (= *Lu. flaviscutellata*) and *Psychodopygus complexus* (Mangabeira, 1941) (= *Lu. complexa*), vectors of *Leishmania* (*L.*) *amazonensis* and *Leishmania* (*V.*) *braziliensis* in the Brazilian Amazonian Region, respectively, exerts an inhibitory effect on parasite infection, resulting in small lesions and low levels of skin parasitism (Francesquini et al. 2014).

In mouse models, Max alone can exacerbate *Leishmania* infection to the same degree as whole salivary glands (Brodie et al. 2007), supporting the idea that it can be applied as a vaccine. In fact, mice vaccinated with Max show marked protection against *Leishmania* infection, producing not only anti-Max antibodies but also producing CD4+ T cells against Max, generating IFN-γ, and inducing NO production (Morris et al. 2001). Immunisation against Max also inhibits blood meal acquisition by sandflies, so immunisation may help block reproduction in the vector (Milleron et al. 2004). Nonetheless, there is Max polymorphism; its amino acid substitution rate is approximately 23%, and some amino acid sequences are not conserved (Warburg et al. 1994, Lanzaro et al. 1999). We may expect to find polymorphisms in Max-simile expressed in *Ny. neivai*.

Patients with ATL previously exposed to Max did not show protection against infection. Anti-Max antibody values were high in patients with the cutaneous and mucocutaneous forms of ATL, with either a positive or negative LST result, either the presence or absence of amastigotes in histopathologic samples, and either positive or negative IIF serology, suggesting that antibodies against Max do not interfere with specific immune responses to ATL. Patients with the aforementioned characteristics have responded well to treatment. In addition, we speculate that exposure to *Ny. neivai* saliva components does not protect a person against infection or prevent the mucocutaneous form of ATL.

While the controls also presented anti-Max antibodies, their values were lower than those in ATL patients. Since the controls live in the same endemic region, we speculate that (i) they were bitten less frequently by sandflies than can be explained by epidemiological and environmental variables such as rural or urban occupation; (ii) they presented strong cellular immune responses ad weak humoral response against Max, resulting in against ATL; or (iii) they are genetically protected against ATL. It would be interesting to have data on the LST response in these individuals. A prospective study with blind evaluators could solve the limitations of our study.

Our data may encourage monitoring of human exposure to Max and to *Ny. neivai* saliva components in ATL endemic regions. Some authors have also shown that vector density and the presence of antibodies against vector saliva are correlated (Clements et al. 2010). Antibodies against Max and antibodies against *Ny. neivai* saliva components may be alternative serological markers to monitor ATL epidemiology in endemic regions. High titres of antibodies against Max in patients with ATL indicate previous exposure to vector bites, and, in our region, they represent previous exposure to the Max-simile peptide of *Ny. neivai*.

Studies on the genetic and protein expression of Max-simile in *Ny. neivai* contribute to the development of new diagnostic approaches, a better understanding of ATL, knowledge of vectors in this endemic region. The examination of anti-Max antibodies in patients with ATL and controls confirms previous exposure to *Ny. neivai* sandfly bites and suggests natural, chronic exposure to vectors. Therefore, antibodies against Max did not protect the host against ATL and could even enhance disease in our region. Further studies are essential to assess polymorphisms and the abundance of Max in *Ny. neivai* salivary glands. A description of the sialotranscriptome would also be of value.
